# The HCV Non-Nucleoside Inhibitor Tegobuvir Utilizes a Novel Mechanism of Action to Inhibit NS5B Polymerase Function

**DOI:** 10.1371/journal.pone.0039163

**Published:** 2012-06-13

**Authors:** Christy M. Hebner, Bin Han, Katherine M. Brendza, Michelle Nash, Maisoun Sulfab, Yang Tian, Magdeleine Hung, Wanchi Fung, Randall W. Vivian, James Trenkle, James Taylor, Kyla Bjornson, Steven Bondy, Xiaohong Liu, John Link, Johan Neyts, Roman Sakowicz, Weidong Zhong, Hengli Tang, Uli Schmitz

**Affiliations:** 1 Gilead Sciences, Inc., Foster City, California, United States of America; 2 Rega Institute, KU Leuven, Rega Institute for Medical Research, Leuven, Belgium; 3 Novartis, Emeryville, California, United States of America; 4 Department of Biological Science, Florida State University, Tallahassee, Florida, United States of America; Scripps Research Institute, United States of America

## Abstract

Tegobuvir (TGV) is a novel non-nucleoside inhibitor (NNI) of HCV RNA replication with demonstrated antiviral activity in patients with genotype 1 chronic HCV infection. The mechanism of action of TGV has not been clearly defined despite the identification of resistance mutations mapping to the NS5B polymerase region. TGV does not inhibit NS5B enzymatic activity in biochemical assays *in vitro*, suggesting a more complex antiviral mechanism with cellular components. Here, we demonstrate that TGV exerts anti-HCV activity utilizing a unique chemical activation and subsequent direct interaction with the NS5B protein. Treatment of HCV subgenomic replicon cells with TGV results in a modified form of NS5B with a distinctly altered mobility on a SDS-PAGE gel. Further analysis reveals that the aberrantly migrating NS5B species contains the inhibitor molecule. Formation of this complex does not require the presence of any other HCV proteins. The intensity of the aberrantly migrating NS5B species is strongly dependent on cellular glutathione levels as well as CYP 1A activity. Furthermore analysis of NS5B protein purified from a heterologous expression system treated with TGV by mass spectrometry suggests that TGV undergoes a CYP- mediated intracellular activation step and the resulting metabolite, after forming a glutathione conjugate, directly and specifically interacts with NS5B. Taken together, these data demonstrate that upon metabolic activation TGV is a specific, covalent inhibitor of the HCV NS5B polymerase and is mechanistically distinct from other classes of the non-nucleoside inhibitors (NNI) of the viral polymerase.

## Introduction

Hepatitis C Virus (HCV) is a major cause of morbidity and mortality with an estimated 170 million people infected worldwide and 3–4 million new infections acquired annually [1]. HCV, a member of the *Flaviviridae* family, is a positive-strand RNA virus with seven major genotypes each divided into a series of subtypes. Additionally, each subtype exists as a quasispecies population within an infected individual [2]. Current treatment utilizing the combination of pegylated interferon (PEG) and ribavirin (RBV) is fraught with significant side-effects and is of limited efficacy against genotype 1 HCV, the most prevalent in the United States and Europe [3], [4]. The recent approvals of HCV NS3 protease inhibitors telaprevir and boceprevir for use in combination with PEG/RBV have significantly improved the effectiveness of the therapy [5], [6]. However, the significant side effects associated with a PEG/RBV-based regimen still remain, and the new antiviral agents have introduced additional tolerability issues. Furthermore, these new treatment options have limited efficacy in certain treatment populations (e.g. PEG-experienced or IL28B *CT/TT* patients) [5], [7], [8]. Recent clinical studies utilizing direct acting antivirals (DAA) in combination suggest that combinations of multiple antivirals with different mechanisms of action and non-overlapping resistance profiles may potentially cure a greater number of HCV patients with shortened treatment duration and even in the absence of PEG and/or RBV [9], [10]. One such agent currently being studied in antiviral combination trials is the non-nucleoside inhibitor (NNI) tegobuvir (TGV).

Tegobuvir (TGV, GS-9190) is an analog of a novel class of imidazopyridine inhibitors selectively targeting HCV [2]. TGV demonstrated anti-HCV potency both *in vitro* and *in vivo*. In a recent exploratory study using TGV in combination with the NS3 protease inhibitor GS-9256 and PEG/RBV, 100% of patients had HCV RNA levels below the lower limit of quantification after only 28 days of treatment [10]. Despite its efficacy and safety in HCV-infected patients, the molecular mechanism of action of TGV has not been well understood. TGV selects (both *in vitro* and in patients) for mutations in the NS5B polymerase at positions 316, 445, 448, and 452 that are responsible for a resistant phenotype [2], [11], [12]. In addition, studies utilizing replicon chimeras demonstrate that TGV potency *in vitro* is linked to NS5B genotype, again indicating that TGV involves the polymerase as a target [2]. However, TGV is not active in biochemical polymerase assays using recombinant NS5B proteins nor could we demonstrate TGV NS5B interactions using various biophysical methods ([2] and unpublished results).

However, these findings can be explained with the novel results presented herein and when considering our recent evidence for the involvement of metabolic activation. Briefly, when co-dosing replicon-harboring cells with different cytochrome P450 inhibitors [2], loss of sensitivity to TGV is observed. This suggests that TGV employs a more complex mechanism of action to target HCV. Here we show that TGV binds directly to the NS5B polymerase after undergoing a unique, multistep metabolic activation pathway that involves specific glutathione adducts.

## Methods

### Replicon cell lines

Huh7-Lunet cells were obtained from ReBlikon GmbH (Mainz, Germany). Creation of Huh7-Lunet cells harboring a stable genotype 1b (Con-1) or 1a (1a H77) replicon encoding a Renilla luciferase reporter has been reported previously [13], [14]. All Huh7-Lunet containing replicon cell lines were grown in Dulbeccos's modified Eagle's medium (DMEM) with GlutaMAX-I (Invitrogen, Carlsbad, CA) supplemented with 10% fetal bovine serum (FBS; HyClone, Logan, UT), 1 U/ml penicillin (Invitrogen), 1 ug/ml streptomycin (Invitrogen) and 0.1 mM nonessential amino acids (Invitrogen). Stable replicon cell lines were maintained in media containing 0.5 mg/ml G418 (Geneticin; Invitrogen). The stable HeLa replicon cell line (clone SL3) was described previously and was obtained from the laboratory of Dr. Christophe Seeger at Fox Chase Cancer Center (Philadelphia, PA) [15]. HeLa replicon cells were grown in DMEM with 10% fetal bovine serum in the presence of 0.5 mg/ml G418.

### Compounds

TGV (GS-9190), VX-222, and compounds 1, 2, and 3 were synthesized at Gilead Sciences, Inc. (Foster City, CA). BILN-2061 and 2′-*C*-methyl adenosine (2′CMeA) were purchased from Acme Bioscience (Belmont, CA). NS5B site IV inhibitor HCV-796 was synthesized by Curragh Chemistries (Cleveland, OH). The benzothiadiazine NS5B polymerase inhibitor (A-782759) was synthesized by ChemALong Laboratories (Lemont, IL).

### Construction of a replicon carrying the Y448H NS5B mutation

A Y448H NS5B point mutation was engineered using the Stratagene Quikchange XL mutagenesis kit (Stratagene) and appropriate primers into the pFK I341 PI-Luc/NS3-3′/ET replicon construct (ReBlikon GmbH) in which the firefly luciferase reporter had been replaced with a Renilla luciferase reporter. Presence of the mutation was confirmed by sequencing (Elim Biopharmaceuticals, Inc., Hayward, CA), after which the NS5B region was then subcloned into a fresh vector backbone using BclI and SpeI restriction sites followed by further sequencing to confirm sequence fidelity. For transfection, DNA plasmids encoding replicon sequences were linearized *in vitro* using SpeI restriction endonuclease (NEB) followed by electrophoresis and gel purification of the linearized fragment (QIAquick gel extraction kit; Qiagen). Replicon RNA was transcribed from the purified template using T7 run-off transcription (MEGAscript T7 kit; Ambion). For transfection of RNA into Huh-7 Lunet cells, cells were trypsinized and washed three times with PBS. A suspension of 4×10^6^ cells in 400 µL PBS was mixed with 10 µg RNA and subjected to electroporation at settings of 270 V and 950 uF capacitance. Cells were then transferred into 20 mL of pre-warmed culture medium and seeded into appropriate plates for further analyses.

### Replicon EC_50_ determinations

Replicon-containing cells were trypsinized and seeded in cell culture media without G418 in white 96-well plates for EC_50_ analysis. Stable replicon carrying cell lines were seeded at a density of 5,000 cells per well. Serial threefold dilutions (10 concentrations) of compounds were performed in DMSO followed by further dilution in cell culture media and subsequent addition to cell plates. Compound-treated cells were incubated 72 hours at 37°C in a 5% CO_2_ incubator. For luciferase-encoding replicons, the luciferase signal (in treated and untreated cells) was quantified using a commercially available assay system (Luciferase Assay System, Promega). For replicons that do not express a luciferase reporter, the antiviral effect was determined using an NS3 protease assay described previously [16]. Curve fitting and EC_50_ values were derived using non-linear regression analysis (XLFit). Curves were extrapolated from duplicate points, and all experiments were individually performed at least twice.

### Western blot analysis

Cells were washed with PBS followed by lysis with RIPA buffer containing protease inhibitors (Complete Mini; Roche) and phosphatase inhibitors (PhoStop; Roche). Samples were placed on ice for 15 minutes then pelleted. Supernatants were snap frozen, and stored in at −80°C. Supernatants were run on 10% Tris-Bis NuPage gels (Invitrogen) in MOPS buffer (Invitrogen) at 200 V for 2 hours under reducing conditions. Gels were transferred to nitrocellulose using the iBlot (Invitrogen) set at 7 minute transfer. Westerns were performed using the SnapID system (Millipore) or the BenchPro system (Invitrogen). Mouse anti-NS5B (clone 3B1) antibody (Axxora), mouse anti-NS5A antibody (Apath), and mouse anti-NS3 antibody (Virostat) were used as primary antibodies for detection of NS5B, NS5A, and NS3, respectively. For Westerns using chemiluminescence analysis, HRP anti-mouse secondary antibody was used (Cell Signaling) and finished blots were incubated briefly with Immunostar Western C reagent (Bio-Rad) then analyzed using a Chemidoc (Bio-Rad). For Westerns using infrared readout, LiCor IR Dye Goat-anti-mouse-800CW was used as a secondary antibody and finished blots were analyzed using the Licor Odyssey (Licor; Lincoln, NE). For detection of biotin, IR Dye 700-linked streptavidin was used.

### BacMam Cloning and Infection

The coding sequences of full-length and 21 C-terminal amino acids truncated NS5B were amplified from the genotype 1b (Con-1) replicon plasmid pFK_i341_PiLucNS3_3′_ET by PCR. The 5′ PCR primer contained a HindIII restriction site, trinucleotide ACC and a methionine codon. The 3′ PCR primer contained coding sequence for a stop codon and an XbaI restriction site. In cases in which a C- or N-terminal His tag were desired, an octahis sequence was also included in the appropriate primer sequence. The amplified fragment was then digested with HindIII and XbaI, and cloned into an analogously digested pFastBacMam vector [17]. Recombinant virus was generated by using the Bac-to-Bac system (Life Technologies, Carlsbad, CA). Virus was further amplified by propagation in Sf21 cells grown in suspension. Sf21 cells used for overexpression of HCV proteins were obtained from Life Sciences (Carlsbad, CA). For expression in hepatocytes, 5×10^6^ Lunet cells were plated in DMEM/10% FBS/NEAA in a T225 flask and incubated overnight in a 37°C 5% CO_2_ incubator. For each flask of cells to be infected, 35 mls of BacMam virus reagent was combined with 70 mL of DMEM/10% FBS/NEAA media and concentrated down to 40 mL using a Vivacell 100 concentrator (Sartorius; MWCO 50,000). Media was removed from Lunet cells and the 40 mls of concentrated BacMam virus was added to each T225 flask. Three hours later, compound was added to the desired concentration as well as sodium butyrate to a final concentration of 10 mM. Flasks were incubated overnight in a 37°C 5% CO_2_ incubator. The next day, the BacMam virus/media mixture from flasks was placed in a 250 mL disposable centrifuge bottle. Cells were washed with PBS, trypsinized, and added to the centrifuge bottle mixture. Cells were pelleted 10 minutes at 1200 rpm, washed with PBS, and pelleted again. Pellets were flash frozen on dry ice and stored at −80°C until purification.

### Purification of NS5B for Mass Spectrometry Analysis

Cell pellets were resuspended and lysed in lysis buffer (50 mM Tris pH 7.5, 350 mM sodium chloride, 2 mM PMSF, 5 mM DTT plus Complete Mini protease inhibitor table). Following lysis, debris was pelleted by centrifugation at 14,000 RPM for 5 minutes at 4°C. Supernatant was incubated overnight with protein A Dynabeads (Life Sciences, Carlsbad CA) previously conjugated to anti-NS5B antibody (Axxora; Clone 12B7). The next day, beads were washed three times with lysis buffer. Captured protein was eluted in 100 mM Glycine (pH 2.4)/4 M Urea and immediate analyzed by mass spectrometry.

### Mass Spectrometry

Mass spectrometry of immunoprecipitated protein samples was performed on an Agilent 6210 Time of Flight Mass Spectrometer with an Agilent 1200 Rapid Resolution HPLC using Masshunter B.02 Acquisition software. The samples were run on an Agilent Zorbax 300 Extend C18 Rapid Resolution column at 70°C, using reverse phase chromatography with a gradient from 20% to 90% acetonitrile containing 0.1% formic acid. Data was processed via Agilent Masshunter B.03 Qualitative Analysis, with Bioconfirm upgrade, allowing for protein deconvolution.

### Cytochrome P450 Overexpression

CYP1A1 DNA was purchased from Open Biosystems (CYP1A1 precision lentiORF) while CYP1A2 (accession NM_00761.3) and CYP3A4 (accession DQ924960.1) DNA were synthesized by Integrated DNA Technologies (IDT). CYP DNA were amplified using 22.5 µL Accuprime pfx supermix (Invitrogen), in reactions containing 0.25 uL of 20 uM forward primer, 0.25 uL of 20 uM reverse primer and 10 ng of DNA. PCR reactions were cycled at 95°C for 5 minutes, then 35 cycles of 95°C for 15 seconds, 57°C for 30 seconds followed by 68°C for 2 minutes. Primer used for amplifications were CYP1A1 forward primer 5′-CAC CGT ACA AAA AAG CAG GCT-3′; CYP1A1 reverse primer 5′-TTA GTA CAA GAA AGC TGG GTC-3′; CYP1A2 forward primer 5′-CAC CAT GGC TCT GTC CCA GTC-3′; CYP1A2 reverse primer 5′-TTA ATT TAT GCT GAA TCT CAA C-3′;

CYP3A4 forward primer 5′-CAC CAT GGC ACT GAT CCC GGA-3′; CYP3A4 reverse primer 5′-TTA GGC GCC GCT CAC GGT GCC-3′. PCR reactions were separated on a 1% agarose gel then purified using the QIAquick Gel Extraction Kit (Qiagen) as per manufacturer's instructions. The purified CYP PCR products were ligated into lentivirus vector (pLenti6/V5-D-TOPO) using pLenti6/v5 directional TOPO cloning kit (Invitrogen) as per manufacturer's instructions and transformed into Stbl3 compentent cells. DNA was extracted from selected colonies and were purified using QIAprep Spin Miniprep Kit (Qiagen) and screened for correct insert size using PCR. Plasmids containing the correct size of insert were confirmed by sequencing (ELIM Biopharmaceuticals; Hayward, CA).

### Transfection of SL3 cells

SL3 cells were plated onto 10 cm cell culture plates at a density of 1.5×10^6^ cells, 24 hours prior to transfection. Each dish of cells was transfected with 15 µg CYP1A1, CYP1A2 or CYP3A4 pLenti6/V5-D-TOPO DNA using 45 µL of TransIT-LT1 (Mirus Bio) according to manufacturer's instructions. Transfection efficiency was determined using RT-PCR to detect expression levels 48 hours post-transfection.

### Quantitative reverse transcriptase polymerase chain reaction (qRT-PCR)

Total RNA was isolated using an RNeasy kit and QIAshredder (Qiagen) according to manufacturer's instructions with DNase digestion. Quantitative analysis of mRNA was carried out on a 7300 Real Time PCR machine (Applied Biosciences). The 25 µL reaction mixture contained 1.25 µL of CYP primer (20×) or 0.25 µL of 10 µM Fam-labeled GAPDH forward primer and 0.25 µL of 10 µM unlabeled reverse primer, 12.5 µL of QuantiTect Multiplex RT-PCR Master Mix (Qiagen), 9.5 µL of nuclease-free water, 0.5 µL of RNA and 0.125 µL of RT-enzyme. Human primers for CYP1A1 (Hs01054797_g1), CYP1A2 (Hs00167927_m1) and CYP3A4 (Hs00604506_m1) were purchased from Applied Biosystems and GAPDH (100H-01) was purchased from Invitrogen. The fold change in the level of CYP1A1, CYP1A2 or CYP3A4 between normal or overexpressed cells were normalized to the levels of GAPDH. The RT-PCR data were analyzed using the relative gene expression method, ΔΔ-Ct.

## Results

### Treatment of HCV replicon containing cells with TGV results in the formation of an NS5B “doublet”

**Figure 1:**
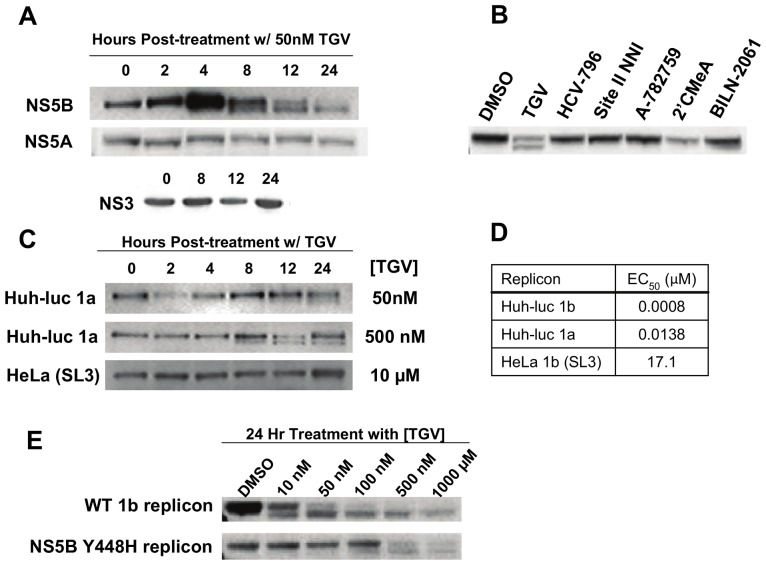
TGV treatment leads to formation of an NS5B double band detectable by Western analysis. A) NS5B, NS5A, and NS3 Western blots of lysates from 1b (Con-1) replicon cells treated with 50 nM TGV for various periods of time. B) NS5B Western blot of lysates from 1b replicon cells treated for 24 hours with known HCV inhibitors at 50× EC_50_ concentrations. C) NS5B Westerns of cell lysates collected at various times from Huh-luc 1a replicon cells treated with 50 nM or 500 nM TGV and lysates from HeLa 1b replicon cells treated with 10 µM TGV. D) TGV EC_50_ values in Huh-luc 1a, 1b, and HeLa 1b (clone SL3) replicon cell lines. E) Western blot of lysates from Lunet cells transiently transfected with wild-type or NS5B Y448H mutant 1b Pi-Rluc replicons and treated with varying concentrations of TGV for 24 hours.

TGV (Scheme S1) is a potent *in vitro* inhibitor of the replication of GT 1a and 1b subgenomic HCV replicons. We first examined what effect TGV treatment of GT 1b replicon cells (1b-Rluc) has on the formation of HCV non-structural proteins by performing Western blot analysis of TGV-treated cultures. Interestingly, treatment of the GT 1b replicon cells with TGV (50 nM; ∼50 fold over EC_50_) resulted in a secondary, faster-migrating NS5B polymerase species by SDS-PAGE such that NS5B was visualized as a doublet by Western blot (Fig. 1. Monitoring of NS5B doublet formation at a fixed concentration of TGV over time revealed that the amount of lower band formed correlated with the time of exposure to TGV. This effect appeared to be specific to NS5B, as no aberration in protein mobility was observed by SDS-PAGE for either NS5A or NS3 proteins after TGV treatment of replicon cells (Fig. 1A). Furthermore, NS5B doublet formation was not observed when replicon-containing cells were treated with other classes of inhibitors including NS5B NNIs (site II inhibitor VX-222, site III inhibitor A-782759 and site IV inhibitor HCV-796), a nucleoside chain terminator (2′CMeA), or a protease inhibitor (BILN-2061), suggesting that this effect is unique to TGV (Fig. 1B).

### NS5B doublet formation correlates with TGV potency

We next explored whether TGV treatment (at 50 nM) also resulted in the formation of a NS5B doublet in GT 1a containing GT 1a replicon cells. Using the same fixed concentration of TGV, the formation of NS5B doublet was examined over time. Although the NS5B doublet was observed in GT 1a replicon cells, the appearance of the lower band was delayed in comparison to GT 1b replicon cells (Fig. 1C). TGV displays sub-genotypic variations in potency such that the GT 1a EC_50_ is 17-fold higher than that observed in GT 1b (Fig. 1D). We increased the concentration of TGV used in the GT 1a replicon 10-fold to determine if increased drug load could overcome the observed delay in doublet formation. A 10-fold higher (500 nM) concentration of drug led to appearance of the lower NS5B band in GT 1a replicon at an exposure time similar to GT 1b, suggesting that doublet formation correlates with TGV antiviral potency (Fig. 1C).

In addition to the sub-type differences in TGV potency, TGV is vastly less potent against the GT 1b (Con-1) replicon in the the HeLa cell line (EC_50_>10 µM). This was unexpected considering the fact that the HeLa replicon was inhibited by all other compounds tested at levels similar to those observed for the 1b-Rluc replicon (data not shown). We wanted to determine if doublet formation occurred in the HeLa replicon following treatment with TGV. Even at the solubility-limited dose of 10 µM of TGV no doublet formation was observed (Fig. 1C). This result further supports the relationship of HCV replication inhibition to doublet formation, as the concentration tested was below the HeLa replicon EC_50_.

Finally, we examined doublet formation in Huh7 cells carrying a replicon harboring a TGV-resistance mutation. NS5B Y448H is the primary mutation conferring both *in vitro* and *in vivo* reduced susceptibility to TGV. GT 1b Y448H NS5B replicons are 36-fold less susceptible [2]. To this end, both wild-type and Y448H GT 1b replicons were transiently transfected into Huh7 cells and cells were analyzed by Western blot analysis for NS5B doublet formation hours following a 24 hr treatment period with varying concentrations of TGV (Fig. 1E). As anticipated, the NS5B lower band was observed in extracts of cells that carry the wild-type replicon and that had been incubated with TGV at a concentration as low as 10 nM. However, the lower band was only faintly visible in extracts of cells that carried the Y448H replicon that had been incubated with 100 nM of TGV. When lower concentrations of TGV were used in the Y448H system no doublet was observed. Taken together, these data indicate that the modified NS5B species observed following treatment with TGV is correlative with TGV activity.

### NS5B doublet formation does not require other viral proteins and is not the result of a cleavage event

**Figure 2:**
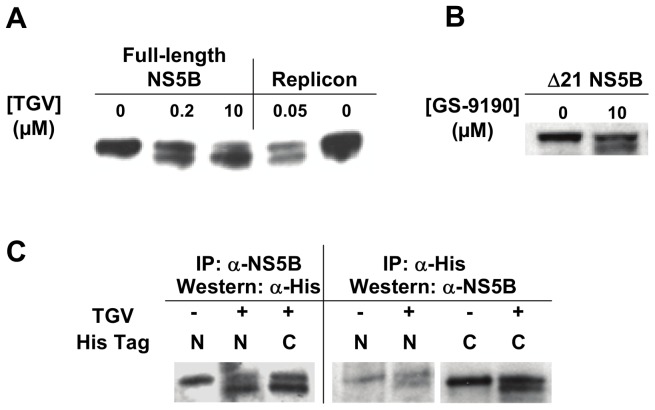
NS5B double band formation in TGV-treated NS5B overexpressing cells. A) NS5B Western blot of lysates collected from TGV-treated Lunet cells expressing full-length NS5B via a BacMam system. Overexpressing cells were treated for 16 hours with various concentrations of TGV and compared to lysates from replicon cells treated for 24 hours with 50 nM TGV or DMSO as a control. B) Western analysis of TGV-treated cells overexpressing a 21 amino acid C-terminally truncated NS5B (Δ21). C) Immunoprecipitation and Western blots of overexpressed Δ21 NS5B encoding an N- or C-terminal His tag. NS5B Western blotting of anti-His immunoprecipitated material and His Western blotting of anti-NS5B immunoprecipitated material are shown.

As the nature of the modified NS5B double band likely held the key to the TGV mechanism of action and the material produced in replicon containing cells proved insufficient for further study, we sought to replicate this phenomenon in a replicon free system. We therefore utilized a BacMam overexpression system to express only GT 1b NS5B polymerase in Huh7 cells. TGV-treated Huh7 cells infected with a full-length NS5B-encoding BacMam virus were examined by Western blot analysis to determine if a NS5B doublet was detectable as observed in the replicon system. As with GT 1b replicon cells, a NS5B doublet was observed in recombinant NS5B expressed using the BacMam system. This demonstrates that other viral proteins are not required to observe this behavior in the presence of TGV (Fig. 2A).

A more soluble deletion construct of NS5B polymerase with the membrane insertion domain removed (Δ21) was expressed using the BacMam system and similarly exhibited doublet formation following TGV treatment (Fig. 2B). Since the lower band of the NS5B doublet migrates 1–2 kDa faster by SDS-PAGE, we hypothesized that this could be due to a deletion at either of the protein termini. Using the NS5B Δ21 construct in the BacMam system, we separately engineered and expressed both N- and C-terminal His-tagged NS5B Δ21. The tagged proteins were expressed in the presence of TGV treatment and immunoprecipitated from cell lysates to determine the nature of the lower band (Fig. 2C). We first performed an immunoprecipitation utilizing an anti-NS5B antibody to pull-down His-tagged NS5B, followed by SDS-PAGE and Western blot analysis using an anti-His antibody. Analysis of the isolated NS5B species revealed that both the N- and C-terminal His tags were intact for both the upper and lower migrating species of the doublet. This indicates that the lower band is not the result of a terminal cleavage. By means of confirmation, the opposite experiment was performed in which the proteins were immunoprecipitated using an anti-His antibody then subjected to Western blotting using an anti-NS5B antibody. Likewise, both NS5B doublet species were formed demonstrating that the N- and C-termini of NS5B are intact in the lower band species and thus that the observed lower band is not the result of protein cleavage or degradation.

### Mass spectrometry reveals an additional higher molecular weight peak in NS5B from TGV-treated cell culture

**Figure 3:**
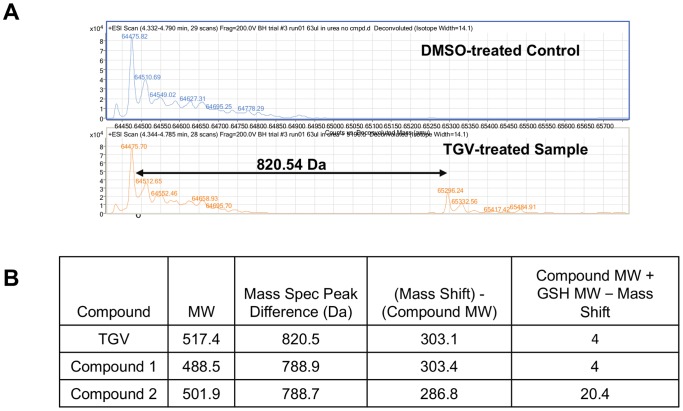
Mass spectrometry identifies higher molecular weight peak in TGV-treated NS5B samples. A) Mass spectrometry analysis of material immunoprecipitated by anti-NS5B antibody from Δ21 NS5B overexpressing cells treated with TGV or DMSO. B) Compound molecular weights and mathematical analysis of resulting peaks detected by mass spectrometry of NS5B samples treated with TGV and analogs.

In lieu of efficient methods to separate the two NS5B species for further characterization, we next employed sensitive biophysical methods to further deduce the nature of the NS5B doublet from the mixture. We immunoprecipitated NS5B from lysates derived from DMSO or TGV-treated BacMam Δ21 NS5B overexpressing cells using an anti-NS5B antibody. Isolated NS5B protein was eluted from the IgG capture beads and immediately analyzed on the mass spectrometer. Analysis of NS5B Δ21 preparation from DMSO treated control samples resulted in a ∼64 kDa peak as anticipated (Fig. 3A). Though NS5B Δ21 isolated from TGV-treated culture contained a similar peak at ∼64 kDa, a second major peak of higher molecular weight was also observed that was unique to the TGV treated sample. Measurement of the molecular weight difference between the two observed peaks resulted in an observed mass change of 820.54 Da. To determine if the additional peak was related to the NS5B Δ21 parent peak, we repeated the experiment with analogs of TGV, compounds 1 and 2 (Scheme S1). In both cases, a second major peak was also observed in the chromatogram, which resulted in mass differences of 788.9 and 788.7 Da, respectively (Fig. 3B). The fact that compounds of different molecular weights can lead to unique peak locations within the deconvoluted spectra suggests that the second observed peak results from binding of the compound to NS5B plus an additional modification. Interestingly, when the molecular weight of the test compounds was subtracted from the mass of the respectively identified second peaks, TGV and compound 1 yielded a difference of 303 Da, while compound 2 resulted in a difference of 286.8 Da. This suggested that the modification did not simply result from the addition of another molecular moiety, but rather a more complex phenomenon dependent on the chemistry of the compound.

### A TGV-related biotinylated compound can be detected in the lower band of NS5B via Western Blot

**Figure 4:**
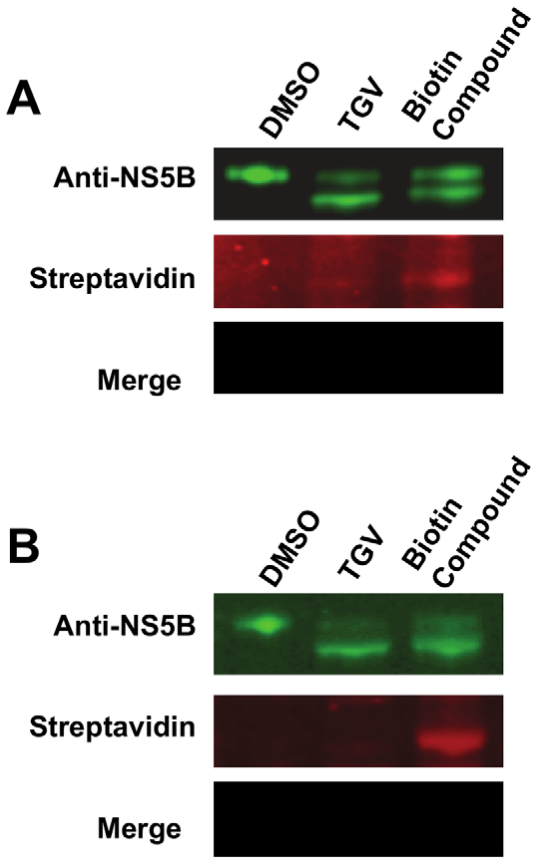
Western analysis of NS5B treated with biotinylated TGV analog shows presence of compound in faster migrating component of double band. A and B Top) Anti-NS5B Western blot of lysates from DMSO-, TGV-, and biotin compound-treated A) Δ21 NS5B overexpressing cells and B) 1b (Con-1) replicon cells. A and B Middle) Fluorochrome-linked streptavidin detection of biotinylated compound 3. A and B Bottom) Merge of NS5B and biotin detection reveals signal co-localization of faster migrating NS5B species and compound.

Prompted by the highly suggestive results from the mass spectrometry experiments, we collected more evidence to confirm that the aberrant migration of TGV treated NS5B is due to compound adducting to NS5B. To this end, BacMam NS5B over-expressing cells were treated with a potent biotinylated TGV analog, compound 3, and lysates of these cultures were subjected to SDS-PAGE (Fig. 4A). Western blot analysis was then performed using a FITC-conjugated secondary antibody for NS5B detection and Texas Red-linked Streptavidin. As with TGV, a NS5B doublet was observed upon treatment with the biotinylated analog. In addition, the Texas Red signal indicating biotinylation was detected in the faster migrating NS5B doublet species of lysates from BacMam over-expressing cells treated with the biotinylated TGV analog, but not in DMSO or non-biotinylated TGV control lysates. We then performed a similar experiment using the 1b-Rluc replicon in Huh-7 cells (Fig. 4B). As observed in the BacMam system, the biotinylated signal was detected in the faster migrating band of NS5B doublet from compound-treated replicon cells, implying that the alternate NS5B species resulting from TGV treatment appears to be due to covalent adduct formation between compound and the NS5B protein.

### Glutathione contributes to TGV activity

**Figure 5:**
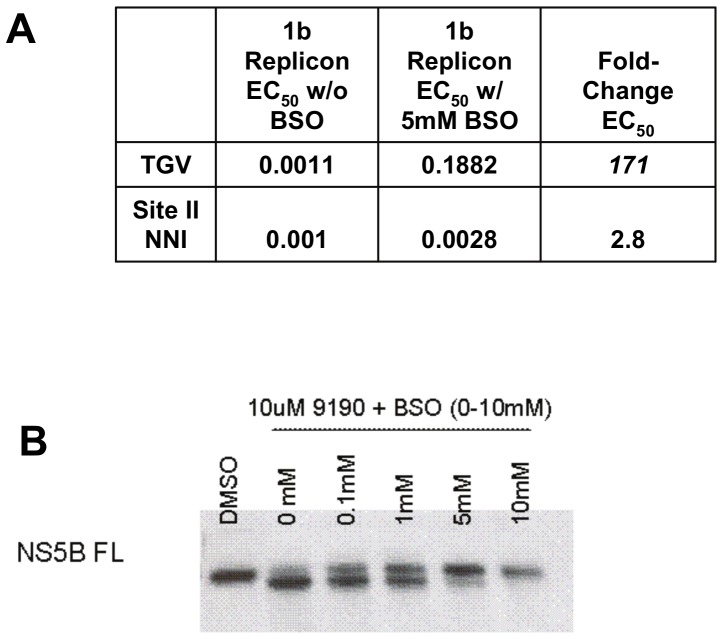
Reduction of GSH by BSO alters TGV potency and NS5B double band formation. A) EC_50_s of TGV and VX-222 in 1b replicon cells co-treated with BSO. Replicon cells were pretreated for 24 hours with 5 mM BSO to reduce GSH prior to compound addition. B) NS5B Western blot of lysates from full-length NS5B overexpressing Lunet cells treated varying concentrations of BSO added together with 10 µM TGV.

Though TGV has been shown to be metabolically very stable (the elimination half-life of TGV in humans is 10–15 hours [18], preclinical metabolite identification studies revealed small amounts of GSH adducts with oxidized versions of TGV (data not shown). In addition, selected cytochrome P450 (CYP) inhibitors were previously shown to alter the antiviral potency of TGV. When TGV was combined in antiviral assays with a CYP1A inhibitor, but not a CYP 3A4 inhibitor, a dramatic decrease in antiviral potency was observed [2]. Taking this into consideration as well as the 820 Da mass difference from the mass spectrometry studies, we surmised that NS5B-TGV adduct formation may result from the oxidative metabolism by CYPs and incorporation of GSH into the complex. To determine the contribution of GSH to TGV activity, we pre-treated replicon cells with L-buthionine sulfoximine, a glutathione synthase inhibitor, to deplete GSH prior to subjecting the GSH-depleted replicons to TGV EC_50_ analysis (Fig. 5A). We observed a 171-fold change in the TGV EC_50_ in BSO-treated replicon cells as compared to control-treated cells. No significant changes in potency were observed utilizing the site II NNI VX-222, suggesting that GSH specifically contributes to TGV activity. As anticipated, NS5B over-expressing cells also demonstrated decreased doublet formation in the presence of TGV when co-treated with BSO (Fig. 5B). Taken together, these data suggest that GSH contributes to TGV antiviral activity.

### CYP 1A1 activity is necessary for the antiviral activity of TGV

**Figure 6:**
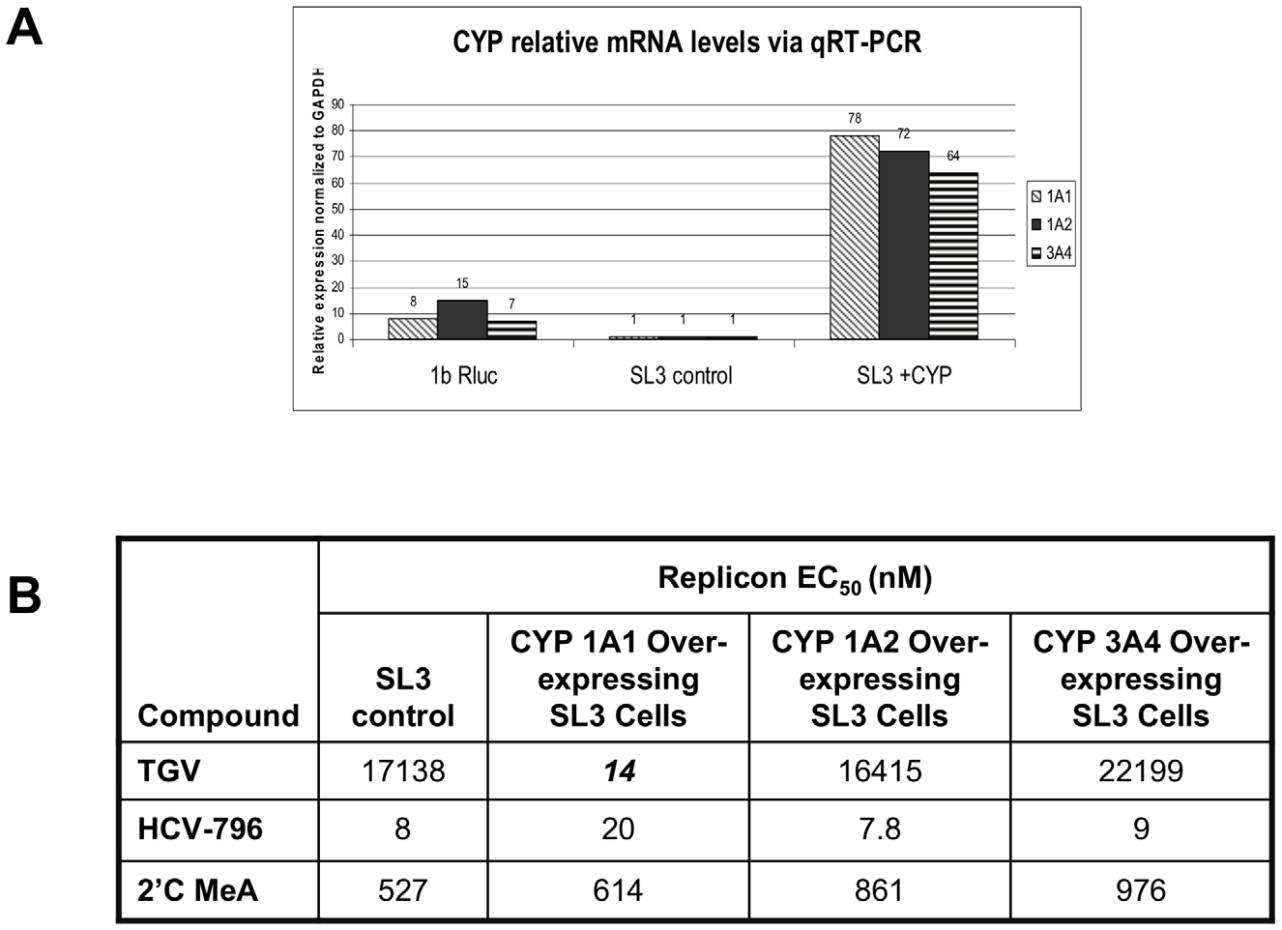
Overexpression of CYP1A1 in SL3 (HeLa GT 1b) replicon cells restores sensitivity to TGV. A) qPCR analysis of CYP1A1, 1A2, and 3A4 mRNA levels in 1b Rluc replicon, control SL3 HeLa 1b replicon cells, and SL3 HeLa 1b replicon CYP1A1-overexpressing cell lines. B) TGV, HCV-796, and 2′CMeA EC_50_s in SL3 control and CYP overexpressing cell lines.

Given the fact that (i) CYP 1A, but not CYP 3A4 inhibitors, result in a decreased antiviral effect of TGV and (ii) the imidazopyridine are the only class of HCV inhibitors with poor anti-HCV activity in the HeLa replicon system (SL3), we hypothesized that this dearth of antiviral effects may be due to low levels of either CYP activity or reduced GSH levels in HeLa cells. GSH levels in HeLa cells were found to be comparable to those in hepatoma cells (data not shown). We then utilized q-RTPCR to gauge the expression of CYPs 1A1, 1A2, and 3A4 in HeLa SL3 versus 1b-Rluc replicon cells. SL3 cells displayed 7- to 16- fold lower expression of CYP mRNA as compared to the Huh-7-based 1b-Rluc replicon (Fig. 6A). We next wanted to determine if we could evoke TGV susceptibility in the SL3 replicon by transiently overexpressing CYPs 1A1, 1A2, or 3A4. The SL3 replicon cells became 1200-fold more sensitive to TGV when overexpressing CYP 1A1, but not 1A2 or 3A4, to reach EC_50_ values similar to those observed in the Huh7 replicon system (Fig. 6B). Note that with the different expression levels of CYP 1A1 tied to TGV activity, it seems conceivable that genotypic variations in antiviral activity can only be referred to the sequence of the replicon if the same host cell line is used. No difference was observed in any of the SL3 cell lines using control inhibitors HCV-796 and 2′CMeA showing that this effect is specific to TGV. These data demonstrate that CYP 1A1 expression levels are essential to TGV activity and suggest that CYP 1A1 metabolism is necessary for TGV to exert an antiviral effect.

## Discussion

TGV is a member of the imidazopyridine class of antivirals which emerged from phenotypic screens [19], [20]. The viral polymerase was established as a molecular target on the basis of reverse genetics. Besides the C316Y mutation near the polymerase active site, several other individual mutations, all in the beta-loop of the thumb domain lead to significant loss in sensitivity to TGV (C445F, Y452H and Y448H) [2]. However, TGV does not inhibit the activity of the HCV polymerase in biochemical assays nor did we find evidence for direct NS5B binding via biophysical methods, i.e. DSC and SPR (data not shown). This apparent discrepancy was largely resolved in light of our recent evidence of the involvement of metabolic activation [2]. In co-dosing experiments with various cytochrome P450 inhibitors, replicon-harboring cells became markedly less sensitive to TGV. Indeed, pan-P450 CYP inhibitors resulted in shifts in the EC_50_ values of TGV. Several, mostly CYP 1A specific inhibitors, proved sufficient to result in this decreased activity. This suggests involvement of a specific oxidative metabolism in the TGV mechanism of action. Such results help explain the lack of conclusive results using more conventional *in vitro* strategies to determine TGV's MOA.

The serendipitous discovery of a NS5B doublet observable by SDS-PAGE provided a marker to experimentally deduce the TGV mechanism of action. From the array of HCV (RdRp) inhibitors studied, only the imidazopyridines result in the formation of the second, faster migrating NS5B band. Moreover, the time at which the band appears first and the size of the band correlate with the potency of the inhibitor. Furthermore, in cells that carry TGV resistant replicons, substantially increased concentrations of drug are needed to result in the formation of a detectable NS5B doublet. Thus the NS5B doublet appears to be a phenotypic hallmark of anti-HCV activity of the imidazopyridine class of compounds.

Due to limitations in the amount of NS5B protein recoverable from the replicon system (Fig. 1A) we utilized a BacMam-based recombinant expression system to produce full-length NS5B as well as the more soluble NS5B Δ21 in large quantities. The signature NS5B doublet was indeed formed when NS5B was produced in the absence of other HCV proteins and treated with TGV. As our NS5B overexpression system could not be optimized to allow analysis of the NS5B doublet bands individually we characterized the NS5B species as a mixture. We quickly ruled out that the 2 kDa faster moving band reflects a TGV-induced protein truncation as both N- and C-termini were found intact by Western blot analysis (Fig. 2C).

To gain further insight into the identity of the second NS5B band, we performed high resolution mass spectrometry on the immunoprecipitated protein fraction containing the NS5B doublet. When analyzing the material obtained from TGV treatment, one additional peak unique to the TGV-treated sample was evident, exhibiting a mass that was 820 Da larger than that of NS5B protein from DMSO-treated cells (Fig. 3A). As this additional mass differs for each inhibitor used for incubation with the overexpression system, it became evident that the inhibitors must be part of the additional mass observed. It came as a surprise that the covalent NS5B-TGV complex migrates faster in a commercial SDS-PAGE system than unmodified NS5B although it has a higher molecular weight. However, SDS-induced denaturation which is generally thought of as a charge mediated unfolding process, may lead to protein species of different compactness, impacting their migration rates. In the NS5B-TGV case, adding a branching point with an additional hydrophobic moiety could lead to a more compact species. Alternatively, the branched hydrophobic moiety may bind disproportionally more SDS which could also yield a faster migration rate.

An important clue for the understanding of the 820 Da difference came from the preclinical metabolite identification studies of TGV and other less metabolically stable analogs using human and dog liver microsomes. One typically observed metabolite of the imidazopyridine analogs showed the addition of GSH along with addition of oxygen and loss of a fluoride ion from the phenyl moiety, corresponding to a MW of 820 in the case of TGV, identical to the NS5B mass shift. The latter shift is 4 Da smaller than the combined molecular weight of TGV and GSH (517+307=824) which can be explained by fluorine/oxygen exchange (Scheme S2). Compounds 1 and 2 (Scheme S1) differ only by the halogen atom on the phenyl moiety. The difference between the sum of compound and GSH MW and the observed mass shift corresponds to the molecular weight difference of the corresponding halogen and oxygen (Fig. 3B; MW HF – MW O=20–16=4 for TGV and compound 1; MW HCl – MW O=36–16=20 for compound 2). This observation led to the hypothesis that an oxidative metabolite of TGV together with GSH covalently modifies the NS5B protein to render it inactive.

Evidence for the involvement of CYP mediated metabolism in the anti-HCV activity of TGV was previously obtained via inhibitor studies [2]. Additional evidence was obtained when CYP 1A1 was overexpressed in the HeLa cells that carry replicons. In HeLa cells TGV does not efficiently inhibit HCV replicon replication. Overexpression of the CYP 1A1 isoform, but not CYP 1A2 or CYP 3A4, restores susceptibility to TGV without affecting the susceptibility to other unrelated NS5B inhibitors (Fig. 6B). Furthermore, the dependence of TGV activity on GSH was established by GSH depletion studies where replicon cells were pretreated with BSO, an inhibitor of the penultimate step of GSH synthesis (Fig. 5B).

Overall, it is clear that oxidative metabolic activation of TGV and normal GSH levels are requirements for TGV to affect antiviral activity. Though our data cannot definitively establish the complete molecular mechanism of TGV, it allows for a plausible explanation for the molecular mechanism of TGV activity as depicted in Scheme S2. Similar to the metabolism of many fluoro-aromatic compounds [21], TGV (Scheme S2, (**1**)) is likely oxidized to an epoxide intermediate (**2**) which can equilibrate with intermediate (**3**). The latter can eliminate the fluoride ion yielding ketone (**4**). The imidazopyridine ring system adjacent to the 2-fluoro-phenyl uniquely allows for a highly stabilized, conjugated intermediate with a double bond between the rings. This creates three different possibilities for a nucleophilic attack by GSH or another reactive cysteine residue (labeled A, B and C on structure (**4**)). In the case of GSH attacking positions A or B, the ensuing adducts (**5a**) and (**5b**) would quickly tautomerize to aromatic, phenol analogs (**6a**) and (**6b**). The latter analogs exhibit molecular weights of 820 Da and correspond to the putative TGV metabolites as discussed above. Pathway C, however, involves an attack at the ipso-carbon of the oxidized phenyl subsituent. This attack would lead to adduct (**7**) which cannot tautomerize to an aromatic phenol structure but is instead prone to a second nucleophilic attack yielding double adducts (**8a**) and (**8b**). The possibility of such a double addition is supported by the observation of minor oxidative metabolites of TGV analogs with two GSH molecules added (data not shown). To explain the NS5B doublet formation, we suggest that reactive intermediate (**4**) undergoes pathway C, with a subsequent addition of GSH and NS5B. The nucleophile of the latter could be the thiol group of an exposed cysteine residue as has been described in earlier reports [22], [23], [24]. In the case of proposed intermediate (**4**) from Scheme S2, it is unclear which addition occurs first; initial GSH addition is probable since GSH is much more abundant than NS5B in the cell.

Although the proposed mechanism of action is plausible, additional evidence for covalent addition of TGV in the replicon system was desired. As several attempts to demonstrate incorporation of radiolabelled compound into the lower band of the NB5B doublet failed, we employed a more sensitive method using the biotinylated TGV analog compound 3 (Scheme S1). The presence of biotin was detected via immunostaining with a streptavidin antibody (Fig. 4) in both BacMam and replicon-derived NS5B preparations. This demonstrates unambiguously that the biotinylated analog is present in the lower band of the doublet obtained from both NS5B preparations.

To summarize, TGV exerts its potent inhibition of HCV replication via a unique and so far unexplored mechanism. Following CYP-mediated oxidative metabolic activation of the fluorophenyl moiety and assistance of GSH, TGV binds covalently to NS5B, thereby disrupting viral replication. This molecular mechanism is unexpected, considering that replicon containing cell lines are typically metabolically compromised and only minuscule amounts of TGV's oxidized GSH-adducts are found in systemic circulation (DMPK data to be published elsewhere). This suggests that TGV's active metabolites react with NS5B with high specificity and efficiency as NS5B should only be present in the liver (the target organ of HCV replication), where GSH levels are high compare to other tissues. When given with pegylated interferon-alfa+RBV or in combination with other direct-acting antivirals, TGV's covalent MOA has not been an impediment for TGV to be generally safe, well-tolerated and associated with potent antiviral activity [25].

## Supporting Information

Scheme S1 **Compounds used in this study.**
(TIF)

Scheme S2 **Proposed chemical mechanism of TGV activiation via CYP mediated oxidative metabolism and involvement of GSH.** Refer to main text for explanation of pathways A–C and molecular species **1–8**.
(TIF)
