# Supplementary figures and images for: The HCV Non-Nucleoside Inhibitor Tegobuvir Utilizes a Novel Mechanism of Action to Inhibit NS5B Polymerase Function
Source: PLoS One. 2012 Jun 13;7(6):e39163. doi: 10.1371/journal.pone.0039163 (PMC3374789; doi:10.1371/journal.pone.0039163)

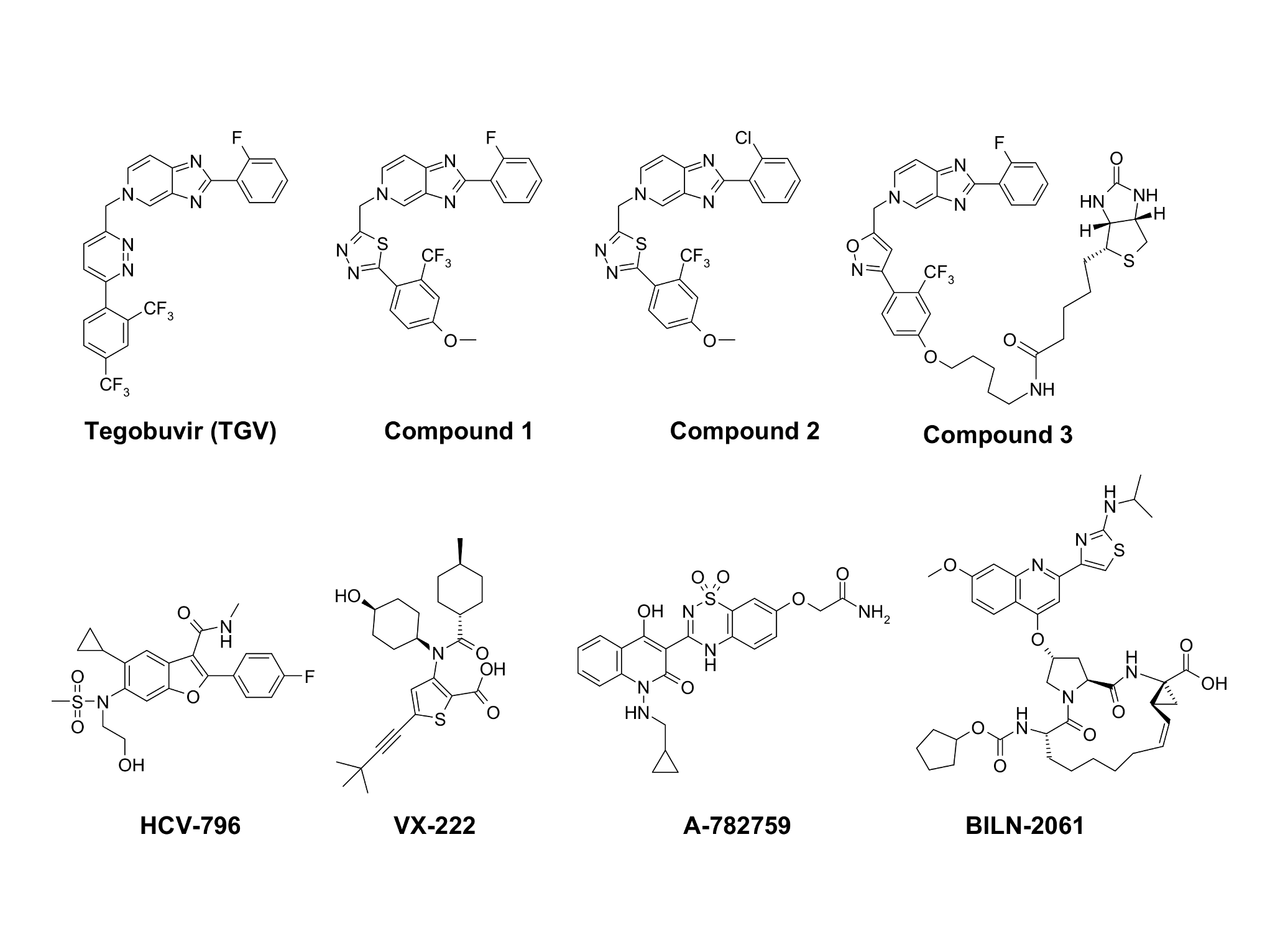

Supplement: Scheme S1 — Compounds used in this study. (TIF) [file pone.0039163.s001.tif]

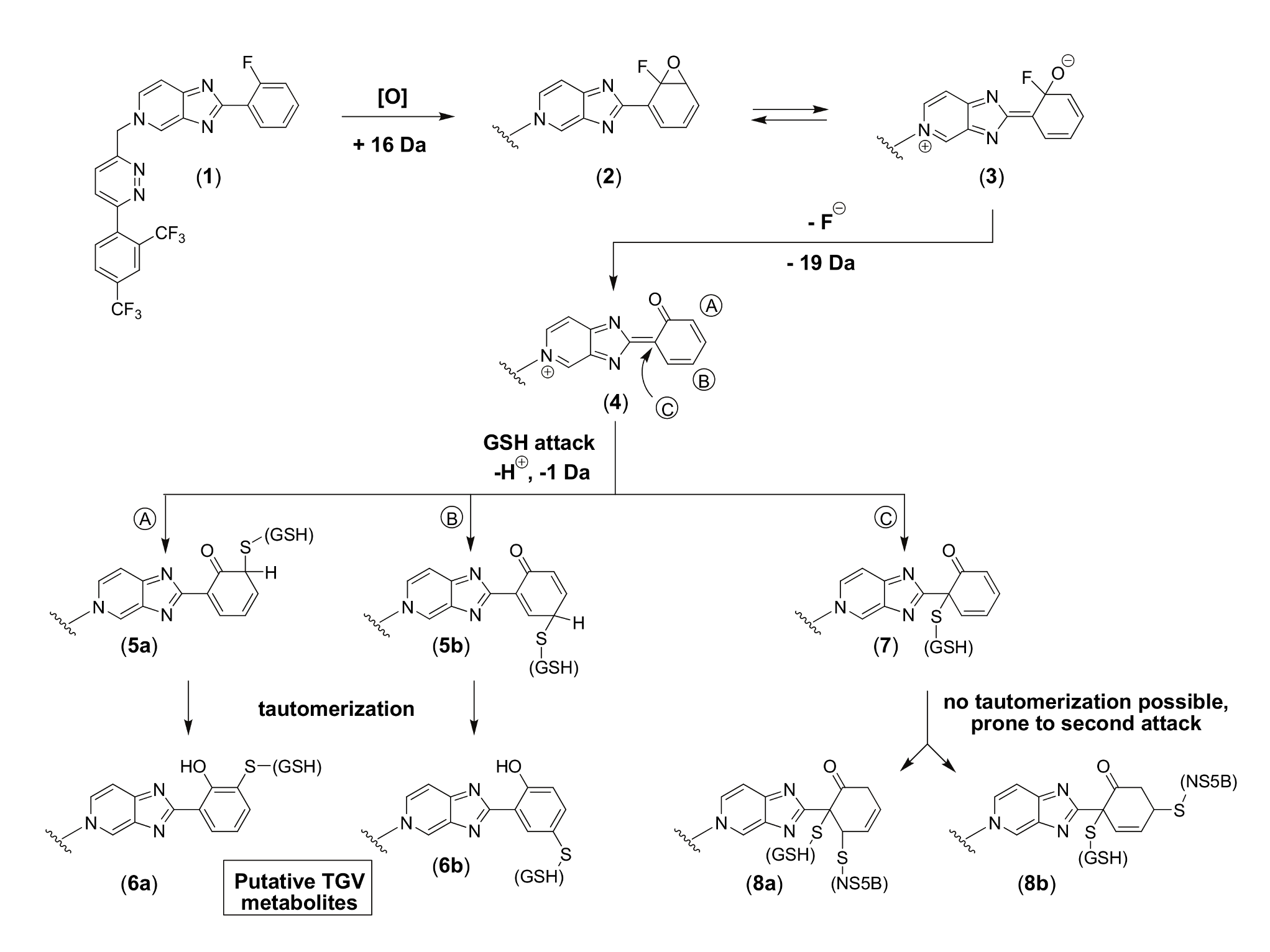

Supplement: Scheme S2 — Proposed chemical mechanism of TGV activiation via CYP mediated oxidative metabolism and involvement of GSH. Refer to main text for explanation of pathways A–C and molecular species 1–8. (TIF) [file pone.0039163.s002.tif]
